# Cholecystitis in older patients following hip fracture: a case series and literature review

**DOI:** 10.1186/s12877-023-04336-9

**Published:** 2023-10-24

**Authors:** Yuan Yuan, Wei Tian, Zhenzhen Jin, Ling Wang, Shiwen Zhu

**Affiliations:** 1 Department of Geriatrics, Beijing Jishuitan Hospital, Capital Medical University, Beijing, 100035 China; 2 Department of Ultrasound, Beijing Jishuitan Hospital, Capital Medical University, Beijing, 100035 China; 3 Department of Radiology, Beijing Jishuitan Hospital, Capital Medical University, Beijing, 100035 China; 4 Department of Orthopedic Trauma, Beijing Jishuitan Hospital, Capital Medical University, Beijing, 100035 China

**Keywords:** Acute cholecystitis, Older, Hip fracture, Mortality

## Abstract

**Objective:**

This study’s aim is to describe the characteristics of perioperative acute cholecystitis in older patients with hip fracture.

**Methods:**

From January 1, 2018, to April 30, 2023, 7,746 medical records were retrospectively collected for patients aged ≥ 65 years who were hospitalised for hip fracture in Beijing Jishuitan Hospital, Capital Medical University. We reviewed 10 cases with confirmed diagnoses of acute cholecystitis.

**Results:**

Of these 10 cases, five femoral neck fractures and five intertrochanteric fractures received orthopaedic surgery. The ratio of males to females was 2:8, the median age was 83.1 years (71–91 years), and there was a median BMI of 25.35 (15.56–35.16). 50% of cases had a poor functional capacity before fracture of below four metabolic equivalents. The median onset time of acute cholecystitis was five days (2–14 days) after fracture, including five cases before orthopaedic surgery and five cases after orthopaedic surgery. All patients had anorexia and fever during the course of the disease. In seven cases of calculous cholecystitis, two underwent percutaneous transhepatic biliary drainage, and one underwent percutaneous cholecystostomy. Two cases of calculous cholecystitis had poor prognosis; one died 49 days after fracture operation, and the reason for death was multiple organ failure caused by severe infection. The other one developed acute cerebellar infarction after gallbladder surgery through treatment in an intensive care unit and neurology department. The case was discharged with dysphasia, and the duration from fracture to discharge was 92 days.

**Conclusion:**

This is the first study on the characteristics of acute cholecystitis in older patients with hip fracture in China. The incidence of acute cholecystitis in our study was 0.13%, with a high risk of in-hospital mortality and elevated hospitalisation costs. Our 10 cases with hip fractures accompanied by acute cholecystitis have common characteristics of poor-to-moderate functional capacity before fracture, increased blood glucose levels and enhanced protein metabolism after fracture. The death and the severe case have similar characteristics of low BMI, multiple underlying diseases, high plasma osmotic pressure and calculous cholecystitis, which occurred after orthopaedic surgery. These issues require attention and prompt, active intervention. Related issues require further research.

## Background

A fragility fracture is a fatal blow to many older people, particularly hip fracture, which is called the ‘last fracture of life’ [1]. In China, the country with the largest population in the world and one that is rapidly becoming an aging society, hip fracture has become a major medical and social problem. A geriatrician-orthopaedics co-management ward was established in our hospital—Beijing Jishuitan Hospital, Capital Medical University—in 2015. Geriatricians and orthopaedic surgeons worked together to manage older patients with hip fracture in order to minimise mortality and improve disease prognosis. Although abdominal complications are rare in patients with hip fracture and hip surgery, the intermittent occurrence of acute cholecystitis prompted us to study the incidence of acute cholecystitis in this particular group of patients. How can we minimise or avoid its occurrence?

Previous studies have revealed that the incidence of acute cholecystitis is 13–50% in people over 70 years of age, and it can be as high as 38–53% in people over 80 years of age [2, 3]. In older patients, acute cholecystitis is more likely to lead to septic shock and, eventually, death [4]. Further, the clinical characteristics of post-traumatic acute cholecystitis are different from those of primary cholecystitis, which has a high missed diagnosis rate and high mortality rate [5]. However, the literature reports on the incidence and disease characteristics of acute cholecystitis in older patients with hip fracture are extremely limited, particularly relevant case reports or studies based in China. As the National Centre for Orthopaedics, we believe that summarising and sharing the clinical characteristics of perioperative acute cholecystitis in older patients with hip fracture in our hospital is representative and necessary.

## Methods

Patients aged 65 years and older who underwent femoral neck or intertrochanteric fracture surgery in our hospital from January 1, 2018, to April 30, 2023, and whose discharge diagnoses included acute cholecystitis were included in this retrospective analysis. All patients followed the same eating and water intake arrangement: fasting and water deprivation after 12 pm the day before the surgery; four to six hours after the surgery, they could try to drink water and consume small amounts of food.

All patients’ data were used anonymously. Informed consent was not necessary for this study due to its retrospective and observational nature. There were 10 cases of acute cholecystitis among 7,746 hospitalised patients who met the age conditions. The female proportion in these 7,746 cases was 70.9% (5,492/7,746). According to the diagnostic criteria of acute cholecystitis, [6] these 10 cases were all confirmed cases. The grading standard is based on the severity assessment criteria for acute cholecystitis: [6] (1) Grade 1 (mild), non-severe, if none of the following serious symptoms are present: elevated white blood cell (WBC) count (> 18,000/mm^3^), palpable tender mass in the right upper abdominal quadrant, duration of complaints > 72 h or marked local inflammation (gangrenous cholecystitis, pericholecystic abscess, hepatic abscess, biliary peritonitis and emphysematous cholecystitis); (2) a patient is considered as Grade 2 (moderate) or intermediate when at least one of the abovementioned conditions are present, but no organ failure has occurred; (3) Grade 3 (severe) is reached in the event of organ failure.

We use metabolic equivalents (METs) [7, 8] to measure and classify the functional capacity of the 10 cases before fracture: (1) poor functional capacity < 4 METs; (2) moderate functional capacity 4–7 METs; (3) good functional capacity > 7 METs.

All cases underwent preoperative anesthesia assessment using the American Society of Anesthesiologists (ASA) physical status classification system (ASAPS) [9]. Classification was as follows: (1) ASA 1 a normal healthy patient; (2) ASA 2 a patient with mild systemic disease; (3) ASA 3 a patient with a severe systemic disease that is not life-threatening; (4) ASA 4 a patient with a severe systemic disease that is a constant threat to life; (5) ASA 5 a moribund patient who is not expected to survive without the operation. The patient is not expected to survive beyond the next 24 h without surgery; (6) ASA 6 a brain-dead patient whose organs are being removed with the intention of transplanting them into another patient.

The related laboratory parameters and their normal ranges are (1) white blood cell (WBC) 3.5–9.5 × 10^9^/L; (2) relative value of neutrophils (NEU%) 40–75%; (3) haemoglobin (HGB) 115–150 g/L; (4) alanine aminotransferase (ALT) 7–40 IU/L; (5) aspartate aminotransferase (AST) 13–35 IU/L; (6) total bilirubin (TBil) 5.1–19 umol/L; (7) direct bilirubin (DBil) 0–7 umol/L; (8) alkaline phosphatase (ALP) 45–125 IU/L; (9) gamma glutamyl transpeptidase (GGT) 7–45 IU/L; (10) c-reactive protein (CRP) 0–8 mg/L; 11) sodium (Na^+^) 135–155 mmol/L; 12) glucose (GLU) 3.9–6.1 mmol/L; 13) blood urea nitrogen (BUN) 3.1–8.0 mmol/L; 14) plasma osmotic pressure (POP) 280–310 mmol/L.

### Statistical methods

SPSS 28.0 (IBM Corp., Armonk, NY, USA) was used in this study to collect data on the listed indicators, as well as to conduct logical verification and management of the data. Descriptive data were expressed through median values (min–max) when in a skewed distribution, and frequency data were expressed as rates.

### Funding

This work was supported by a grant from the National Natural Science Foundation of China (Code: 82,072,445).

## Results

### Basic information features

There were eight women (8/10: 80%) and two men (2/10: 20%) with a median age of 83.1 years (71–91 years) and a median BMI of 25.35 (15.56–35.16). Five patients were overweight (BMI > 25). The BMI of the death case (case 8) was the lowest, followed by the case 5, then the severe case (case 2). Poor functional capacity (< 4 METs) before fracture was seen in 50% of cases (cases 1, 7–10); moderate functional capacity (4 ~ 7 METs) was seen in 50% of cases (cases 2–6).

Eight of the 10 patients had chronic underlying diseases, including hypertension in four cases (cases 3, 4, 8, 10); among these cases, cases 4 and 8 were complicated with old cerebral infarction. Coronary heart disease was found in cases 2 and 9, including case 2 with diabetes and case 9 with old cerebral infarction and chronic lung disease. Chronic lung disease combined with dementia was found in case 1, and chronic renal insufficiency was found in case 7. No case had undergone abdominal surgery or major surgery, and none had a history of tumours.

### Perioperative conditions

Five patients with femoral neck fracture received femoral head replacement, and five patients with intertrochanteric fracture received intramedullary nail fixation. The preoperative anaesthesia assessment was from Grade 1 to 4. Except for general anaesthesia in case 9, all other patients underwent neuraxial anaesthesia. The durations of the surgeries were 40–90 min; the intraoperative blood loss was 100–400 mL, and the intraoperative blood transfusion volume was 0–400 mL. Further, the median time from fracture to surgery was 6.5 days (2–14 days), and the median time from admission to orthopaedic surgery was two days (1–7 days).

Among the 10 patients, the median time from fracture to acute cholecystitis was five days (2–14 days), which included five cases (cases 1, 6, 7, 9, 10) before orthopaedic surgery and five cases (cases 2–5, 8) after surgery, as presented in Table 1.

**Table 1:** Basic characteristics of the 10 cases with acute cholecystitis during the perioperative period of hip fracture

Case	Age	Gender	BMI	METs before fracture	Types of fracture and surgery	ASA classification	Surgery duration (min)	Blood loss/ transfusion during operation (ml)	Fracture to surgery (day)	Admission to surgery (day)	Fracture to onset (day)	Surgery to onset (day)
1	71	F	23.51	< 4	Femoral neck Replacement	3	60	200/0	14	7	8	-6
2	75	F	22.58	4–7	Femoral neck Replacement	2	50	100/0	8	1	14	6
3	80	F	25.97	4–7	Femoral neck Replacement	3	70	100/400	2	2	3	1
4	83	F	31.25	4–7	Trochanter Internal fixation	2	60	100/0	3	1	5	2
5	83	F	17.15	4–7	Femoral neck Replacement	3	90	200/400	3	2	4	1
6	85	F	28.23	4–7	Femoral neck Replacement	1	90	100/0	6	3	3	-3
7	87	M	35.16	< 4	Trochanter Internal fixation	2	85	200/400	6	2	5	-1
8	90	F	15.56	< 4	Trochanter Internal fixation	4	40	400/400	7	2	11	4
9	91	M	31.11	< 4	Trochanter Internal fixation	4	60	100/0	13	3	10	-3
10	86	F	23	< 4	Trochanter Internal fixation	3	60	200/400	8	5	2	-6

Notes: BMI, Body Mass Index; METs, metabolic equivalents

### Clinical signs and symptoms

All patients had different degrees of anorexia and fever during the course of the disease. The median maximum body temperature was 37.8℃ (37.6℃–38.5℃), in which the highest body temperatures of the cases 1, 3, 8 and 9 were all higher than 38℃. The median duration of fever was three days (1–7 days), in which the fever processes of case 1 and case 9 reached seven days. Further, there were seven cases who had abdominal pain (cases 2–6, 8, 9), five cases who had nausea (cases 2, 3, 4, 9, 10), two cases who had vomiting (cases 4 and 9) and six cases who had Murphy’s sign (cases 2–6, 8).

### Laboratory tests and severity evaluation

The median time from fracture to the first blood test was 2.5 days (0–9 days). Three patients had gastrointestinal symptoms on the day of the first test (case 6 had fever and epigastric pain; case 9 had nausea and vomiting with fever; case 10 had nausea and fever), and the remaining seven patients had no gastrointestinal symptoms or fever at the time of the first test.

Table 2 indicates that cases 6, 9 and 10 had gastrointestinal discomfort on the day of the first test, and their WBC, CRP, ALT and AST were also significantly higher than normal, which was consistent with their symptoms. Eight cases experienced varying degrees of anaemia throughout the entire course of the disease (cases 1, 3, 4–8, 10), in which case 7 had HGB below 90 g/L. Although case 1 had increased ALT, AST, GGT and ALP, there was no obvious gastrointestinal symptom and no increase in body temperature at the first visit. In case 7, WBC and CRP did not increase significantly after the onset of acute cholecystitis, but there was a significant increase in the level of hepatobiliary enzyme.

**Table 2:** Laboratory results of the 10 patients before symptoms and in the phase of acute cholecystitis

Case	Period	WBC	NEU%	HGB	ALT	AST	TBiL	DBiL	GGT	ALP	CRP	Na+	GLU	BUN	POP
1	Pre-symptom Acute phase	9.79 11.72	70.6 88.5	115 103	243 211	228 174	10.9 9.0	3.1 2.9	144 155	165 142	101.8 27.7	139 143	9.2 9.9	7.6 7.2	303 310.7
2	Pre-symptom Acute phase	8.67 26.13	77.7 91.1	149 118	15 19	14 36	16.9 20.8	4.1 12.5	36 45	78 103	68.08 291.25	138 129	13.2 10.2	11.6 3.7	311.8 298.9
3	Pre-symptom Acute phase	13.46 10.12	92.5 87.1	113 97	16	17	34.7	12.3	12	48	< 1.0 202	135 133	8.6	8.7	295.9
4	Pre-symptom Acute phase	9.08 19.02	72.6 91.3	111 121	13 118	15 143	12.3 41.5	3.2 27.3	7 564	48 413	49 180	140 137	7.4 14.7	5.8 6.0	300.4 301.5
5	Pre-symptom Acute phase	16.19 33.06	90.4 96.5	128 114	14 249	23 206	14.3 378.4	5.7 229.8	7 116	42 77	3 27.8	136 137	6.8 10.8	5.0 10.5	291.4 301.3
6	Symptoms day Acute phase	13.25 14.82	86.3 88.6	118 114	128 167	186 41	39.8 43.1	19.1 22.6	141 275	72 162	127.6 171	135 132	7.1 7.0	5.9 5.3	291.6 281.5
7	Pre-symptom Acute phase	6.51 4.28	81.5 80.9	70 90	14 134	26 66	26.1 207.1	11.2 131.4	12 119	32 92	138.78 23.58	137 141	8.2 7.2	24 13.7	314.4 311.9
8	Pre-symptom Acute phase	14.98 24.47	84.7 96.3	101 111	17 62	23 58	34.8 179.4	10.1 63.9	8 114	35 175	28.8	139 136	7.3 3.4	19.5 5.2	312.6 286.8
9	Symptoms day Acute phase	13.82 13.26	84.6 82.5	128 125	101 99	56 169	20.7 22.6	9.3 9.5	94 75	150 152	140 105	142 140.5	17 10.1	17.7 15.0	306.6 300.54
10	Symptoms day Acute phase	11.47 8.56	83.5 78.2	91 92	83 53	53 27	30.4 52.1	10.1 18.6	29 30	82 74	93.3 41.2	141 138	10.5 8.5	10.8 9.0	312.1 307.3

Note: WBC, white blood cell; NEU%, Neutrophils %; HGB, hemoglobin; ALT, alanine aminotransferase; AST, aspartate aminotransferase; TBil, total bilirubin; DBil, direct bilirubin; GGT, gamma glutamyl transpeptidase; ALP, alkaline phosphatase; CRP, C-reactive protein. Na^+^, sodium; GLU, glucose; BUN, blood urea nitrogen; POP, plasma osmotic pressure [POP (mmol/L) =([Na^+^] + [k^+^]) +Glucose/18+BUN/2.8(mmol/l)].

As shown in Table 2, no patients were found to have hypernatremia at the first visit and the onset of acute cholecystitis. Cases 2 and 3 showed hyponatremia at the onset of acute cholecystitis. Six patients (cases 2, 3, 7–10) had an increase in BUN at their first visit, and 100% of patients had a random venous blood glucose level higher than 6.1 mmol/L. The values of POP at these two time points showed that a mild increase occurred in cases 1, 2, 7, 8 and 10 during the course of the disease, and no patients with decreased POP were found.

**Table 3:** Ultrasound, CT and treatment findings in the 10 older patients with hip fracture complicated with acute cholecystitis

Case	Abdominal ultrasound				Abdominal CT				Grade classification	Treatment *	Other acute complications	Transfer status/prognosis
	Gallbladder enlargement	Wall thickening/irritability	Gallstone	Others	Gallbladder enlargement	Wall thickening/irritability	Gallstone	Others				
1	No	No	No	No	No	Yes	No	No	2	Parenteral nutrition Cefotaxime Sodium and Sulbactam Sodium ivgtt 10 days		No/discharged
2	Yes	No	Yes	Gallbladder fossa effusion	Yes	No	Yes	No	2	***Cholecystostomy*** Fasting and water deprivation Parenteral nutrition Levofloxacin ivgtt 10 days	Acute cerebral infarction	***General surgery-ICU-neurology department***/discharged
3					No	Yes	No	No	2	Cefotaxime Sodium and Sulbactam Sodium ivgtt 3 days	Lower GI hemorrhage	No/discharged
4	Yes	Yes	Yes	No	Yes	No	Yes	Perihepatic effusion	2	Fasting and water deprivation Parenteral nutrition Cefotaxime Sodium and Sulbactam Sodium ivgtt 7 days	Incomplete intestinal obstruction	Geriatrics department/discharged
5	Yes	Yes	Yes	Calculus of upper segment of extrahepatic bile duct	Yes	No	Yes	No	2	***Biliary drainage*** Fasting and water deprivation Parenteral nutrition Cefotaxime Sodium and Sulbactam Sodium ivgtt 6 days	Acute obstructive jaundice	ICU-another hospital for continued treatment of cholecystitis/discharged
6	Yes	No	No	No					2	Cefotaxime Sodium and Sulbactam Sodium ivgtt 6 days	Deep vein thrombosis of the lower extremities	Vascular surgery department (retrievable inferior vena cava filter removal)/discharged
7	Yes	No	No	No	Yes	No	Yes	Gallbladder fossa effusion	2	Fasting and water deprivation Parenteral nutrition Moxifloxacin ivgtt 8 days		ICU/discharged
8	Yes	No	No	No	Yes	No	Yes	Choledocholithiasis	3	***Biliary drainage*** Fasting and water deprivation Parenteral nutrition Imipenem and cilastatin sodium ivgtt 8 days	Acute cholangitis	**ICU/died**
9	Yes	Yes	Yes	No	Yes	Yes	No	No	2	Fasting and water deprivation Parenteral nutrition Imipenem and cilastatin sodium ivgtt 6 days		No/discharged
10	Yes	Yes	Yes	No					1	Cefotaxime Sodium and Sulbactam Sodium ivgtt 7 days		No/discharged

* including first-choice antibiotics and the course of treatment

### Imaging characteristics

**Fig. 1:**
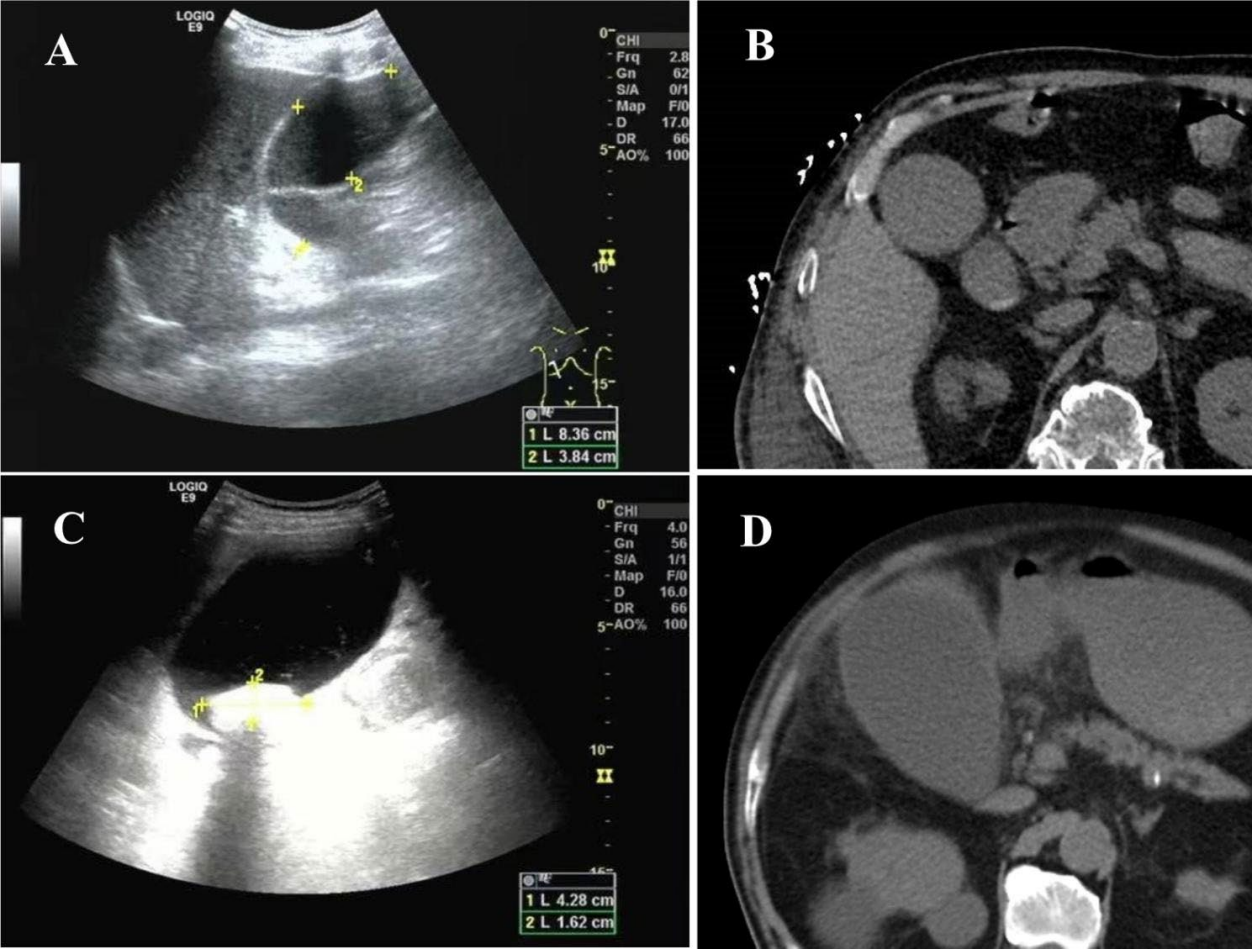
Abdominal ultrasound and CT Findings in two cases **A**. The ultrasound results of case 7 revealed that there was gallbladder enlargement with no stones. **B**. The abdominal CT of case 7 revealed the presence of gallstones. **C**. In case 9, abdominal ultrasound revealed gallstones with gallbladder enlargement. **D**. In case 9, no stones were found in the abdominal CT.

Table 3 indicates that during the onset of acute cholecystitis, seven patients (cases 1, 2, 4, 5, 7–9) underwent abdominal ultrasound and abdominal computer tomography (CT) simultaneously, and the consistency rate of gallbladder stone examination was 57.14% (4/7) (cases 1, 2, 4, 5). Of the four cases in which the detection results of two examinations were consistent, only case 1 was the non-calculous type. For case 3, only abdominal CT was performed, and for cases 6 and 10, only abdominal ultrasound was performed. No gallbladder and bile duct stones were found in either case; therefore, the non-calculous type cannot be excluded. Figure 1 shows the inconsistency between the above two examinations in the detectability of gallbladder stones in cases 7 and 9.

### Treatment

Table 3 indicates that six patients (cases 2, 4, 5, 7–9) were given fasting and water deprivation for a median of 4.5 days (1–5 days), and seven patients (cases 1, 2, 4, 5, 7–9) were given parenteral nutrition support for a median of seven days (2–12 days). All 10 patients were treated with intravenous anti-infective therapy, with a median duration of seven days (3–10 days). Six of them were given third-generation cephalosporins (cases 1, 3–6, 10), two were given carbapenems (cases 8, 9) and two were given quinolones (cases 2, 7) as the first-choice antibiotics. Further, two patients (cases 5, 8) underwent bile duct drainage on the diagnosis day of acute cholecystitis and the third day after the diagnosis, respectively. Case 2 underwent cholecystostomy on the third day after the diagnosis of acute cholecystitis.

### Prognosis and costs

Table 3 indicates that of the 10 patients, two cases had poor prognoses, of which case 2 was transferred from the orthopaedics department to the general surgery department for gallbladder surgery. They developed acute cerebellar infarction on the day after the gallbladder surgery and were transferred to an intensive care unit because of a consciousness disorder, then to the neurology department for treatment. Thereafter, case 2 was discharged with dysphasia in a stable condition, and the duration from fracture to discharge was 92 days. The other case (case 8) died in the intensive care unit due to multiple organ failure induced by severe infection with a time period from fracture to death of 54 days (49 days after surgery). Case 4 was transferred to the geriatric department and discharged from the hospital thereafter. Case 5 was transferred from the intensive care unit to another hospital for treatment of acute cholecystitis. The mortality of older patients with hip fracture and the complication of acute cholecystitis (1/10, 10%) was much higher than that of older patients without acute cholecystitis (16/7,736, 0.2%) during the same period, and the gender ratio of these 16 deaths was 8:8.

Further, the median time from admission to final discharge or death (including hospitalisation days in other departments) was 10.5 days (5–92 days). The median hospitalisation cost of the ten patients was 72,663.13 CNY (46,983.94–385,186.34 CNY), which was much higher than that of the 7,736 hip fracture patients without acute cholecystitis in the same ward and during the same period, whose median cost was 52,231.85 CNY (9,121.8–900,511.9 CNY).

## Discussion

The characteristics of acute cholecystitis in older Chinese patients with hip fracture have not been reported yet. This study shows that the incidence of perioperative acute cholecystitis in older patients with hip fracture in our hospital was 0.13% (10/7,746); the mortality was 10% (1/10), which was much higher than the all-cause mortality (0.2%, 16/7,736) of hip fracture patients without cholecystitis in the same ward and during the same period and also higher than the mortality rate of older people with simple biliary tract infections reported in some studies (6.1%, 35/574) [10]. Similar to the results of our study, one cohort study from the Korean National Health Insurance Service-Senior, [11] in which 15,210 hip fracture patients were enrolled, showed that 36 patients developed acute cholecystitis within 30 days after the hip fracture date (30-day cumulative incidence, 0.24%), and 11.1% of acute cholecystitis patients (4/36) died within 30 days. Another Korean study reviewed 10 years of medical records on hip fracture and found that the incidence of developing cholecystitis within two months after hip fracture surgery was 0.74% (9/1,211) [12]. The incidence rate of this study was higher than in ours, which was considered to be related to the longer follow-up time.

In terms of gender differences, there are research reports [13, 14] that acute cholecystitis has higher incidence in women and relatively worse prognosis and higher cost in men. Older hip fracture also has higher incidence in women and relatively more complications and higher mortality in men [15]. A study from South Korea [11] showed that older patients with hip fracture complicated by acute cholecystitis were mainly female (66.6%, 24/36), while the gender ratio of the deceased four patients, who died within 30 days after surgery, was not specified. In our study, the proportion of women with acute cholecystitis was higher (8:2), but both critically ill and deceased patients were female. The correlation between gender and disease prognosis requires an analysis of larger sample data to verify.

A cohort study [16] showed that older female patients with poor nutritional conditions, including low serum albumin levels, low BMI, vague symptoms and no abdominal pain might have delayed cholecystitis diagnosis. The death case in this study had low METs and low BMI before fracture, although clinical symptoms included abdominal pain without nausea or vomiting, so that the disease condition is also easily overlooked or delayed.

This study showed that, at the first visit for hip fracture, 100% of cases had blood glucose levels higher than the upper limit of normal (ULN) fasting blood glucose level, 60% had BUN higher than the ULN, and 40% had POP higher than the ULN. The main mechanism was increased endogenous gluconeogenesis, decreased oxidative utilisation of glucose in tissues and organs and increased insulin resistance and protein breakdown in peripheral tissues caused by traumatic stress. The severe case (case 2) and the death case (case 8) in this study had hyperglycaemia, high BUN and elevated POP, indicating that these may play a role in inducing disease progression. Simultaneously, the incidence of acute cholecystitis after surgery was more severe (cases 2, 3, 4, 5, 8), which may be related to re-stress caused by multiple factors such as fasting and water deprivation before hip surgery and surgical stress.

At the same time, it is also necessary to consider the possibility of gallbladder arteriosclerosis in older patients, which might cause gallbladder mucosal erosion or ischemic necrosis during acute inflammation, dehydration or trauma phases. Therefore, for older patients diagnosed with arteriosclerosis, it may be more beneficial to continue to take antiplatelet drugs orally during the perioperative period for hip fracture. The safety of antiplatelet drugs during the perioperative period has been confirmed, and they are widely used in our ward [17].

Imaging examination is currently one of the main methods for the diagnosis of acute cholecystitis [6]. The most commonly used modalities are ultrasound and CT examination. As the preferred examination method, the sensitivity and specificity of ultrasound in the diagnosis of acute cholecystitis were 81% and 83%, respectively [18]. Compared with ultrasound, abdominal and pelvic CT appears to be more sensitive and specific, particularly in the diagnosis of lesions in the bile duct—a score from a study conducted in 2012 presents sensitivity > 83% and a specificity of approximately 83% in acute cholangitis diagnosis, irrespective of the cause [19]. In this study, for cases 7 and 8, bile duct stones were not identified by ultrasound examination, but the CT results were positive for stones. However, in case 9, a gallbladder stone was identified by ultrasound, while the CT results were negative. Thus, it is suggested that in clinical practice, multiple examinations are often required for a more accurate determination of the condition.

In this study, one case was definitely non-calculous, and two cases were suspected non-calculous (only ultrasound or CT examination did not find stones). The proportion of non-calculous acute cholecystitis was higher than the 5–10% found in the general population [20, 21]. The confirmed case of the non-stone type had abnormal laboratory indicators before the appearance of clinical symptoms, thereby suggesting that patients with acute cholecystitis may present with delayed clinical symptoms and, thus, delayed onset of the disease. Some studies reported that the course of non-calculous acute cholecystitis was more complex, and the mortality rate was higher; [20, 22] however, the symptoms of the three patients in our study were relatively mild. On the contrary, the conditions of the patients with calculous acute cholecystitis were relatively severe, and one of these patients who underwent biliary drainage died. The relatively mild clinical course of non-calculous cholecystitis in this study may be due to the following reasons: (1) timely detection and early treatment: two of the three cases were detected before surgery, and one case was detected on the first day after surgery—early detection ensured timely intervention; and (2) the patients were relatively young.

## Conclusion

This study has limitations. First, our study was a single-centre study, and the representativeness of the sample was insufficient. Due to these shortcomings, the number of cases of acute cholecystitis was insufficient, and additional statistical analyses such as related risk factors could not be performed. Second, the incidence of acute cholecystitis after hip fracture in older patients is largely underestimated because patients with hip fracture first go to the emergency department of a hospital; a proportion of the patients with acute cholecystitis after hip fracture are usually diverted to the department of gastrointestinal surgery. Simultaneously, due to the influence of retrospective analysis, patients’ perioperative intake and postoperative recovery times cannot be obtained, and whether intervention measures such as shortening the preoperative fasting time and increasing the volume can effectively reduce the occurrence and development of acute cholecystitis still needs to be verified in further research.

In conclusion, although the incidence of perioperative acute cholecystitis in older patients with hip fracture in this study is low, it leads to a significant increase in hospital mortality and medical costs during hospitalisation. The death case had low BMI, low METs, high ASA grade, calculous cholecystitis and cholecystitis occurring after orthopaedic surgery. Meanwhile, patients with non-calculous cholecystitis may have inconsistency between symptoms and laboratory tests, which should be heeded to avoid delays in cholecystitis diagnosis and treatment.

## Data Availability

With the permission of the corresponding authors, we can provide participant data without names and identifiers. The corresponding authors have the right to decide whether or not to share the data based on the research objectives and plan provided. Please contact the corresponding authors for data requests.

## Abbreviations

US: Ultrasound
CT: Computer tomography
METs: Metabolic equivalents
WBC: White blood cell
HGB: Haemoglobin
ALT: Alanine aminotransferase
AST: Aspartate amino transferase
TBil: Total bilirubin
DBil: Direct bilirubin
ALP: Alkaline phosphatase
GGT: Gamma glutamyl transpeptidase
CRP: c-reactive protein
Na^+^: Sodium
GLU: Glucose
BUN: Blood urea nitrogen
POP: Plasma osmotic pressure
